# Antibody Repertoires Identify β-Tubulin as a Host Protective Parasite Antigen in Mice Infected With *Trypanosoma cruzi*

**DOI:** 10.3389/fimmu.2018.00671

**Published:** 2018-04-13

**Authors:** Fabricio Montalvão, Danielle Oliveira Nascimento, Marise P. Nunes, Carolina M. Koeller, Alexandre Morrot, Leticia Miranda S. Lery, Paulo M. Bisch, Santuza M. R. Teixeira, Rita Vasconcellos, Leonardo Freire-de-Lima, Marcela F. Lopes, Norton Heise, George A. DosReis, Célio Geraldo Freire-de-Lima

**Affiliations:** ^1^Faculdade de Medicina de Petrópolis (FMP-FASE), Petrópolis, Brazil; ^2^Instituto de Biofísica Carlos Chagas Filho, Universidade Federal do Rio de Janeiro, Rio de Janeiro, Brazil; ^3^Laboratório de Imunoparasitologia, Instituto Oswaldo Cruz, Fundação Oswaldo Cruz, Rio de Janeiro, Brazil; ^4^Faculdade de Medicina, Universidade Federal do Rio de Janeiro, Rio de Janeiro, Brazil; ^5^Laboratório de Microbiologia Celular, Instituto Oswaldo Cruz, Fundação Oswaldo Cruz, Rio de Janeiro, Brazil; ^6^Departamento de Bioquímica e Imunologia, Universidade Federal de Minas Gerais, Belo Horizonte, Brazil; ^7^Instituto de Biologia, Universidade Federal Fluminense, Niterói, Brazil

**Keywords:** *Trypanosoma cruzi*, antibody repertoires, beta-tubulin, lymphocyte activation, Chagas disease

## Abstract

Few studies investigate the major protein antigens targeted by the antibody diversity of infected mice with *Trypanosoma cruzi*. To detect global IgG antibody specificities, sera from infected mice were immunoblotted against whole *T. cruzi* extracts. By proteomic analysis, we were able to identify the most immunogenic *T. cruzi* proteins. We identified three major antigens as pyruvate phosphate dikinase, Hsp-85, and β-tubulin. The major protein band recognized by host IgG was *T. cruzi* β-tubulin. The *T. cruzi* β-tubulin gene was cloned, expressed in *E. coli*, and recombinant *T. cruzi* β-tubulin was obtained. Infection increased IgG reactivity against recombinant *T. cruzi* β-tubulin. A single immunization of mice with recombinant *T. cruzi* β-tubulin increased specific IgG reactivity and induced protection against *T. cruzi* infection. These results indicate that repertoire analysis is a valid approach to identify antigens for vaccines against Chagas disease.

## Introduction

Chagas disease is caused by the protozoan parasite *Trypanosoma cruzi* and imposes a heavy burden on human health in Latin America. Infection with *T. cruzi* leads to parasitemia and spread of the parasite to host tissues. Control of *T. cruzi* infection depends on cells of innate and acquired immunity, which produce cytokines, inflammatory mediators, and antibodies (1, 2). However, tissue infection persists indefinitely at low levels (1, 2). Several studies indicate the importance of antibodies in protection against *T. cruzi* infection (3, 4, 5, 6), but the precise role of humoral immunity in host defense remains incompletely understood.

Purified *T. cruzi* proteins induce protection in mice challenged with live parasites. These antigens include cruzipain (7), *trans*-sialidase (8), members of mucin-associated surface protein family (9), and excretory-secretory antigens (10). However, most studies amplify detection by using recombinant antigens, or probing with antibodies from immunized animals. New studies have characterized novel parasites antigens that could be targeted in vaccine studies (11, 12). An alternative approach to the testing of randomly purified molecules is the identification of *T. cruzi* antigens which are targeted by the host antibody diversity in the course of infection. Antibody diversity can be analyzed by an immunoblot technique which detects global antibody reactivity against whole protein extracts (13). This method detects autoantibodies produced in autoimmune diseases (14, 15) and identifies repertoire changes linked to resistance against *T. cruzi* infection (16).

Microtubules are cytoskeletal structures composed of α/β tubulin heterodimers that are found in eukaryotic cells and are abundant in trypanosomatid parasites from the order Kinetoplastide (17). These structures have important functions in cell division, maintenance of cellular morphology, motility, intracellular transport, and signal transduction (18). In trypanosomes microtubules have two α-tubulin isoforms and one β-tubulin isoform (19) and are found underneath the plasma membrane (the subpellicular microtubules), in the flagellum, and as a component of mitotic spindle apparatus (20). The microtubules function as a perfect target for many compounds with trypanocidal activity, blocking tubulin activity (21). Therefore, a microtubule component is a suitable target to be considered as an effective vaccine candidate to protect from trypanosome infections (22, 23). It was previously shown that mice vaccinated with native tubulin purified from *Trypanosoma brucei* were protected against *T. brucei, Trypanosoma congolense*, and *Trypanosoma rhodesiense* infection (22). Previous report showed that mice vaccinated with the microtubule-associated protein (MAP) p52 of *T. brucei*, together with aldolase, GAPDH, and MAP p15, were protected after challenge with a homologous infection (24). Furthermore, rabbit antibodies to tubulin-rich fractions from *T. brucei* inhibit the growth of trypanosomes in culture (25).

Given the paucity of information regarding the subject, this work addresses the identification of prominent *T. cruzi* antigens targeted by IgG antibodies during infection. Here, we describe that acute and chronic infection of BALB/c mice induced limited changes in the antibody diversity. Using a proteomic approach, we identified *T. cruzi* β-tubulin (TcβTUB) as one of the major antigens targeted by antibodies. The *T. cruzi* β-tubulin gene was isolated, cloned, and expressed. Recombinant TcβTUB was recognized by sera from infected mice, and immunization of naïve mice with a single dose of recombinant TcβTUB induced protection against infection. These results indicate the importance of selecting candidate vaccines antigens from analysis of unbiased Ab reactivities from infected mice.

## Materials and Methods

### Mice, Parasite, and Infection

Male wild-type (WT) BALB/c and Fas-L mutant BALB/c.*gld* (*gld*) mice aging 6–8 weeks, weighing 25–30 g were from the Oswaldo Cruz Institute Animal Care facility, Rio de Janeiro. BALB.*gld* mice (26) were produced at the National Institutes of Health, Bethesda, MD, USA by serially backcrossing the *gld* gene onto a BALB/c background for 15 generations.

All mouse studies followed the guidelines set by the National Institutes of Health, United States. The study was approved by the Research Ethics Committee of Federal University of Rio de Janeiro (protocol 062/14). Protocols for animal were approved by the Institutional Ethical Committees in accordance with international guidelines. All animal experimentation was performed in accordance with the terms of the Brazilian guidelines for the animal welfare regulations.

Mice were infected with intraperitoneal injection (i.p.) with 10^5^ chemically induced metacyclic forms of *T. cruzi* clone Dm28c (17, 18). Chemically induced and insect-derived metacyclic forms induce a similar infection as demonstrated previously (18). Acute infection was evaluated after 23–33 days of infection, while chronic infection was evaluated after 150 days.

### Recombinant *T. cruzi* β-Tubulin

Genomic DNA was extracted from *T. cruzi* Dm28c epimastigotes (1 × 10^8^; Rapidprep isolation kit, Pharmacia) and used as template for amplification of *T. cruzi β-tubulin* gene (19) by touchdown PCR (27). Touchdown PCR was carried out in 50 µL of 20 mM Tris–HCl (pH 8.8), 2 mM MgSO_4_, 10 mM KCl, 10 mM (NH_4_)_2_SO_4_, 0.1% (v/v) Triton X-100, 0.1 mg/mL nuclease-free BSA, 100 ng DNA, 0.5 mM dNTPs, 0.4 µM of primers TcβTubF (5′-ATCATATGCGTGAGATTGTGTGCG) and TcβTubR (5′-ATGAATTCTTAGTACTGCTCCTCCTC), and a mixture of 2.0 U Pfu (Fermentas) and 0.25 U Taq (Biotools) DNA polymerases. The resulting 1,342 bp fragment was cloned into pTZ57R/T (Fermentas), sequenced and sub-cloned, yielding pET-28a-TcβTUB expression plasmid. pET-28a-TcβTUB was introduced into BL21 (DE3) *E. coli* strain, and transfectants were induced with 0.5 mM isopropyl 1-thio-β-d-galactopyranoside (Invitrogen, USA) for 18 h at 20°C. Cells were suspended in 300 µg/mL lysozyme (Sigma) in 50 mM Tris–HCl (pH 7.5), 200 mM NaCl, 5% (w/v) glycerol, 1 mM DTT, and protease inhibitor mix (Sigma), and left 30 min on ice. After addition of 10 U DNAse I (Fermentas) and 5 mM MgCl_2_, the suspension was incubated for 30 min on ice and lysed by sonication. The lysate was centrifuged at 30,900 *g* for 40 min at 4°C, and SDS-PAGE indicated the presence of recombinant *T. cruzi* β-tubulin (TcβTUB). The pellet was solubilized in 2% Triton X-100, 2 M Urea, 100 mM Tris–HCl (pH 7.5), 5 mM Na_2_EDTA, 5 mM DTT, 5 mM imidazole, and protease inhibitor mix. The supernatant containing TcβTUB was applied onto a His-Trap HP (GE Healthcare) column pre-equilibrated with 2 M Urea, 100 mM Tris–HCl (pH 7.5), 0.5 M NaCl containing 5 mM imidazole (wash buffer). Recombinant TcβTUB was eluted with wash buffer containing 0.25 M imidazole, analyzed for purity on SDS-PAGE, and quantified by the method of Bradford (28) using BSA as standard.

### ELISA

For determination of serum IgG, microplates (Greiner BiOne) were coated with 2 µg/mL goat anti-mouse IgG (Southern Biotechnology) for 16 h at 4°C. Concentrations were calculated based on standard curves generated with purified mouse IgG (Southern). In addition, microplates were coated with dsDNA from calf thymus, histone from calf thymus, myosin from rabbit heart, KLH (5 µg/mL; all from Sigma), or *T. cruzi* antigen diluted at 1:1,000. *T. cruzi* antigen was obtained by lysing epimastigotes in buffer containing 200 mM Tris, 400 mM NaCl, 40 mM EDTA, 20 mM iodoacetamide, and 1 mM PMSF. ELISA tests for IgG against recombinant TcβTUB (0.5 µg/mL) were performed. Plates were blocked with PBS-1% gelatin (Vetec, Brazil) for 1 h and washed with PBS containing 0.05% Tween-20. Sera were kept at −20°C until use. Data show the results with 1:100 serum dilution. Binding was determined following incubation with alkaline phosphatase-conjugated goat anti-mouse IgG (Southern). Reaction was developed with PnPP substrate (Southern) diluted in Tris–MgCl_2_ buffer. Absorbance was read at 405 nm.

### Immunoblots

For antibody repertoire analysis, mouse hearts were lysed in extraction buffer (2% SDS, 5% 2-mercaptoethanol, and 62.5 mM Tris, pH 6.8) on ice, without protease inhibitors, at a proportion of 1 g/10 mL buffer. *T. cruzi* epimastigotes (clone Dm28c) were lysed in 10 mL extraction buffer (5 × 10^6^/mL). Extracts were sonicated for 10 min, boiled for 10 min, centrifuged at 1,000 *g*, and then at 10,000 *g*. Supernatants were stored at −20°C. IgG reactivities against heart and *T. cruzi* polypeptides were identified by a modified immunoblot technique (13). Briefly, extracts (600 µg/mL) were subjected to SDS-PAGE, and proteins were transferred to nitrocellulose. Membranes were blocked for 18 h with PBS-0.2% Tween-20 at RT and incubated for 4 h with sera adjusted for 100 µg/mL IgG concentration, using the Cassette Miniblot System (Immunetics, Cambridge, MA, USA). Alkaline phosphatase-conjugated secondary goat anti-mouse IgG antibody (Southern) was added for 90 min. After washing, immunoreactivities were revealed with nitroblue-tetrazolium/bromo-chloro-indolyl-phosphate (NBT/BCIP; Promega) and analyzed by densitometry. Blotted proteins were stained with colloidal gold (Bio-Rad) and subjected to a second densitometry. The immunoblot and protein scans were superimposed and rescaled to correct migration irregularities. Adjusted profiles were divided into sections representing an IgG reactivity. Section reactivities were quantified as the average optical density expressed as peak values and subjected to multiparametric statistical analysis (13). Data analysis was performed on an iMac computer using the Igor software (WaveMetrics, Lake Oswego, OR, USA) (13, 16). The statistical test employed comparison between curves, rather than individual bands. Densitometric profiles represent the mean of 5–8 individual sera. Immunoblots against recombinant TcβTUB were performed with either anti-mouse β-tubulin III monoclonal antibody 5G8 (Promega) or anti-β-tubulin rabbit monoclonal antibody 9F3 (Cell Signaling). Anti-α-tubulin monoclonal antibody B-5-1-2 (29) was used as control. Mouse brain extract (MBE) (25 µg protein/lane) was used as source of mouse tubulin (30). Blots were incubated with 1:10,000 Alexa-680-labeled anti-mouse IgG (Invitrogen) or IRDye-800-labeled anti-rabbit IgG (Li-Cor), diluted in TBS with 3% BSA for 1 h at RT. Fluorescence was imaged using an Odyssey^®^ infrared scanner (LI-COR Inc.). Blots were scanned and processed using Adobe Photoshop CS and Image J 1.43m software.

### Mass Spectrometry

*Trypanosoma cruzi* extracts were subjected to SDS-PAGE in parallel mirror lanes. One lane was blotted and reacted with serum to identify the major bands. Following precise location, the mirror lane was excised in the gel. Excision and processing of protein spots were performed as previously described (31). Digestions were done with sequencing grade porcine trypsin (Promega, Madison, WI, USA) at 37°C for 16 h. Tryptic peptides (0.5 mL) were mixed with a saturated solution of CHCA matrix in 50% ACN, 1% TFA (0.5 mL), spotted onto an MALDI sample plate, and allowed to crystallize at room temperature. MALDI/TOF-TOF peptide sequencing, operated in reflectron-delayed extraction mode with high resolution for the 800–4,000 Da range, was performed by precursor ion fragmentation, using N_2_ gas in the collision cell at 2.8 × 10^−6^ torr, in a 4700 Explorer Proteomics Analyzer (Applied Biosystems). Protein identification was carried out against the NCBI database using the MASCOT software (www.matrixscience.com) and the following parameters: *cys*-carbamidomethylation as fixed modification; methionine oxidation as variable modification; one missing trypsin cleavage, monoisotopic masses, peptide, and ion tolerances at 0.2 Da. Under these conditions, a probability based score 81 was considered significant (*p* < 0.05). The other criteria for identification were a minimum of 20% of protein coverage and four peptides with hits in the database (for PMF data) or two peptide sequence tags (for MS/MS).

### Immunization Assays

BALB/c mice were injected with PBS, BSA, or recombinant TcβTUB (both 20 µg/animal) emulsified in Complete Freund Adjuvant (CFA, Thermo Scientific) in the hind footpads. After 14 days, mice were infected with 10^5^ metacyclic trypomastigotes as above. Parasitemia was evaluated by tail vein puncture; viable parasites were counted in a Neubauer chamber. Mice were sacrificed at day 32 of infection, and individual spleens were weighted. Individual sera were collected and analyzed by western blotting for reactivity against *T. cruzi* and mouse tubulins.

### Statistical Analysis

Data were analyzed by Student’s *t*-test for independent samples and ANOVA test, using SigmaPlot™ for Windows. Data from parasitemia were normalized by log transformation before statistical testing. Data from densitometric analysis were analyzed by Mann–Whitney test, using Graph Pad InStat 3.01 for Windows. Differences with a *p* value < 0.05 or lower were considered significant.

## Results

### Antibody Reactivities Against Exogenous and Autologous Antigens in *T. cruzi* Infection

**Figure 1:**
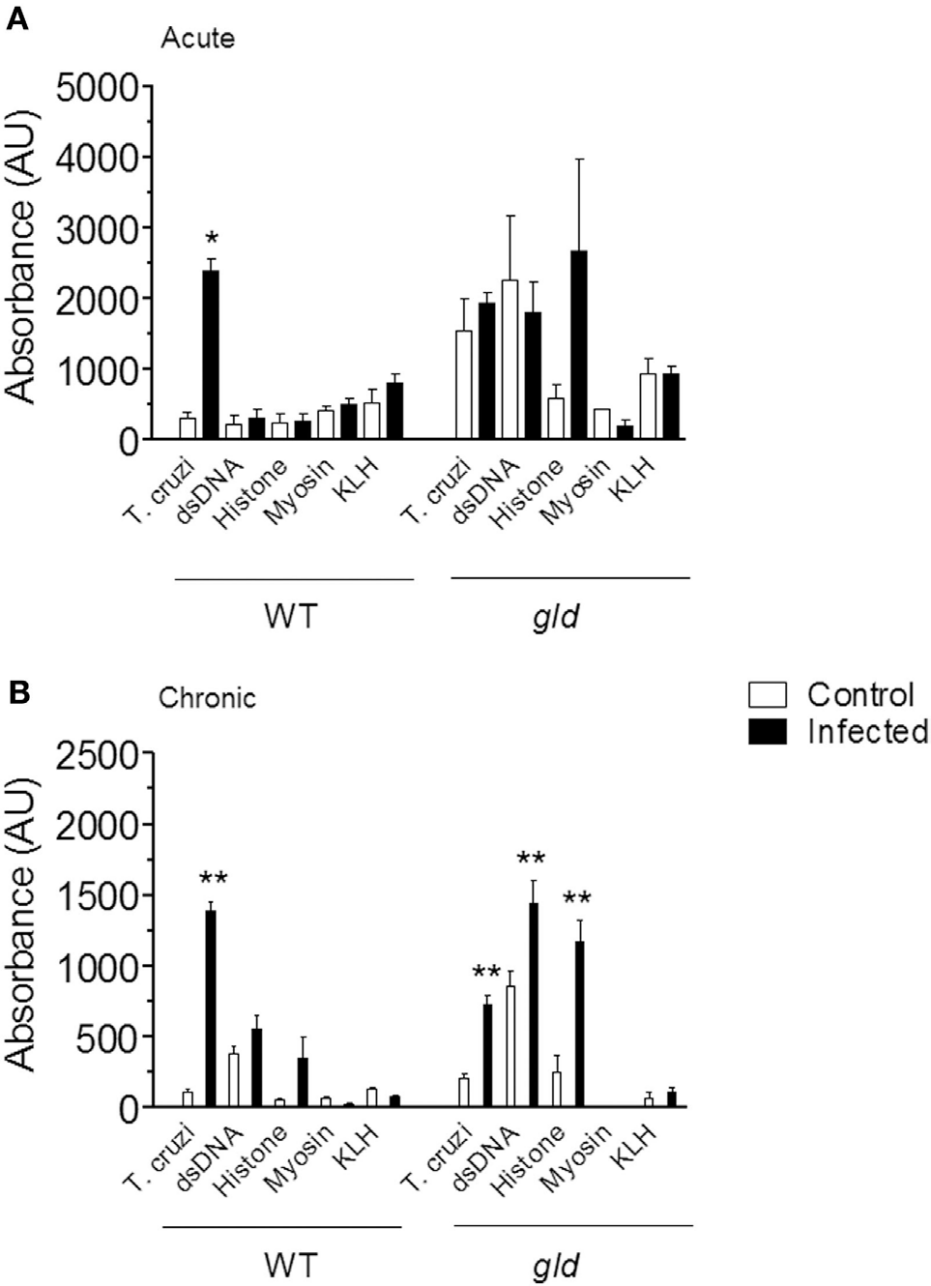
Analysis of serum IgG specificities in wild-type (WT) and *gld* BALB/c mice during acute **(A)** and chronic **(B)** infection with *Trypanosoma cruzi*. Serum IgG specificities from control (open bars) and infected mice (closed bars) were assayed by ELISA on plates coated with the indicated antigens. All sera were diluted 1:100. Data are mean and SEM of 3–12 mice per group. Statistical analysis was performed by *t*-test from representative results of three similar experiments (*p < 0.05; **p < 0.01).

We infected BALB/c mice with chemically induced metacyclic forms of *T. cruzi* clone Dm28c (17, 18). Chemically induced and insect-derived metacyclic forms induce a similar infection (18). Acute and chronic infection with *T. cruzi* increased the concentrations of serum IgG (data not shown). We measured IgG reactivities against a panel of exogenous and autologous antigens (*T. cruzi*, KLH, myosin, dsDNA, and histone) by ELISA in the sera of control and infected mice. Only IgG reactivities against *T. cruzi* antigens increased after acute (Figure 1A, left) or chronic infection (Figure 1B, left). As a positive control for autoreactivity, we used sera from lupus-prone *gld* mice for comparison (Figures 1A,B, right). Our results show that a highly focused Ab response against *T. cruzi* antigens, and not autoreactivity, is the final outcome following infection.

### Global Analysis of IgG Reactivities Against *T. cruzi* and Cardiac Polypeptides

**Figure 2:**
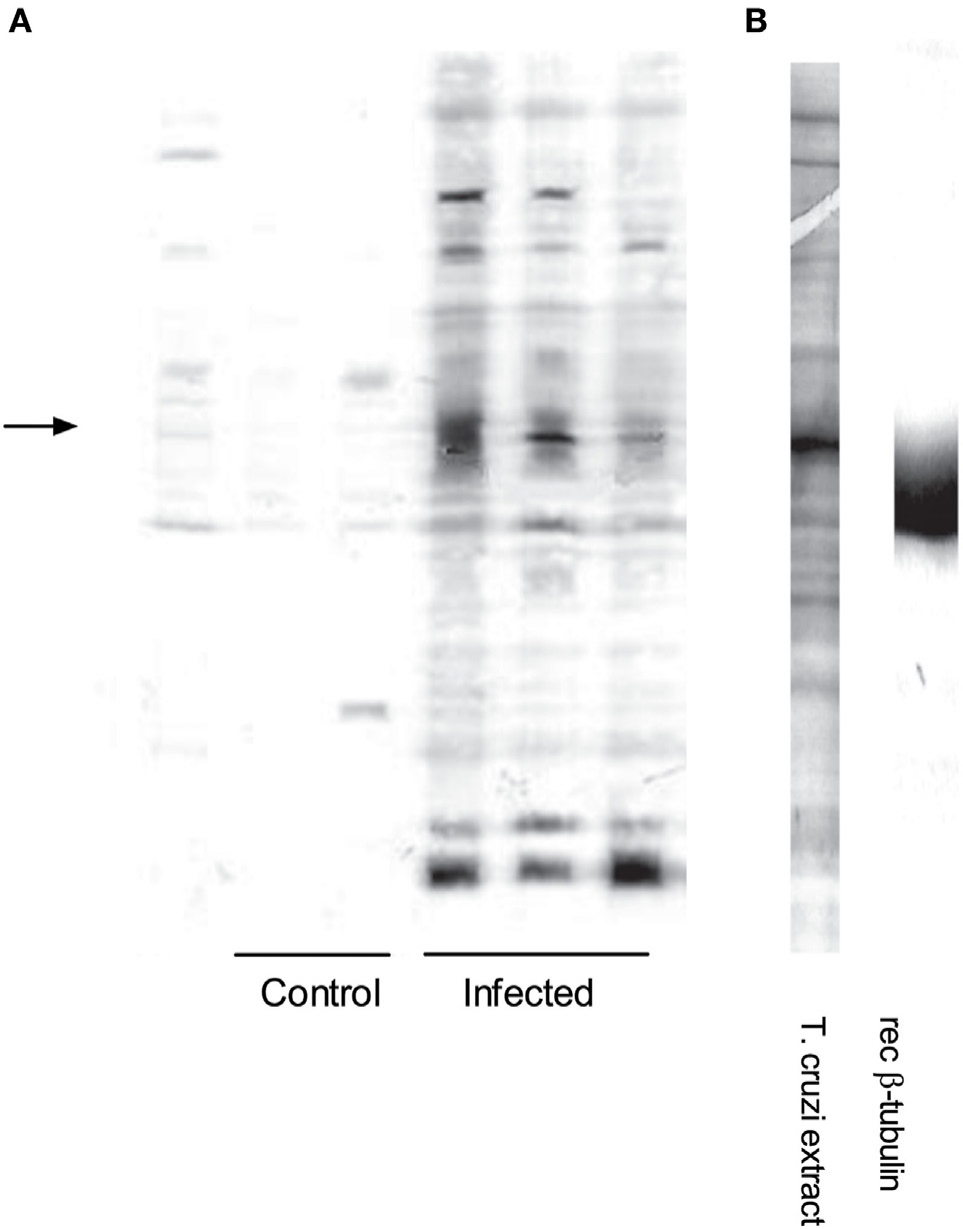
Infection induces a distinct antibody repertoire: a major reactivity elicited against *Trypanosoma cruzi* is directed to *T. cruzi* β-tubulin. **(A)** Immunoblot showing individual serum IgG reactivities from control (*n* = 3) and chronically infected BALB/c mice (*n* = 4) against *T. cruzi* extracts. The left arrow indicates the major 50–55 kDa protein band in the protein extract. IgG concentrations of all sera were adjusted to 100 µg/mL. **(B)** Left lane: Coomassie blue staining of SDS-PAGE showing the protein profile of *T. cruzi* extract. **(B)** Right lane: immunoblot of recombinant TcβTUB probed with anti-mouse β-tubulin III monoclonal antibody 5G8.

**Figure 3:**
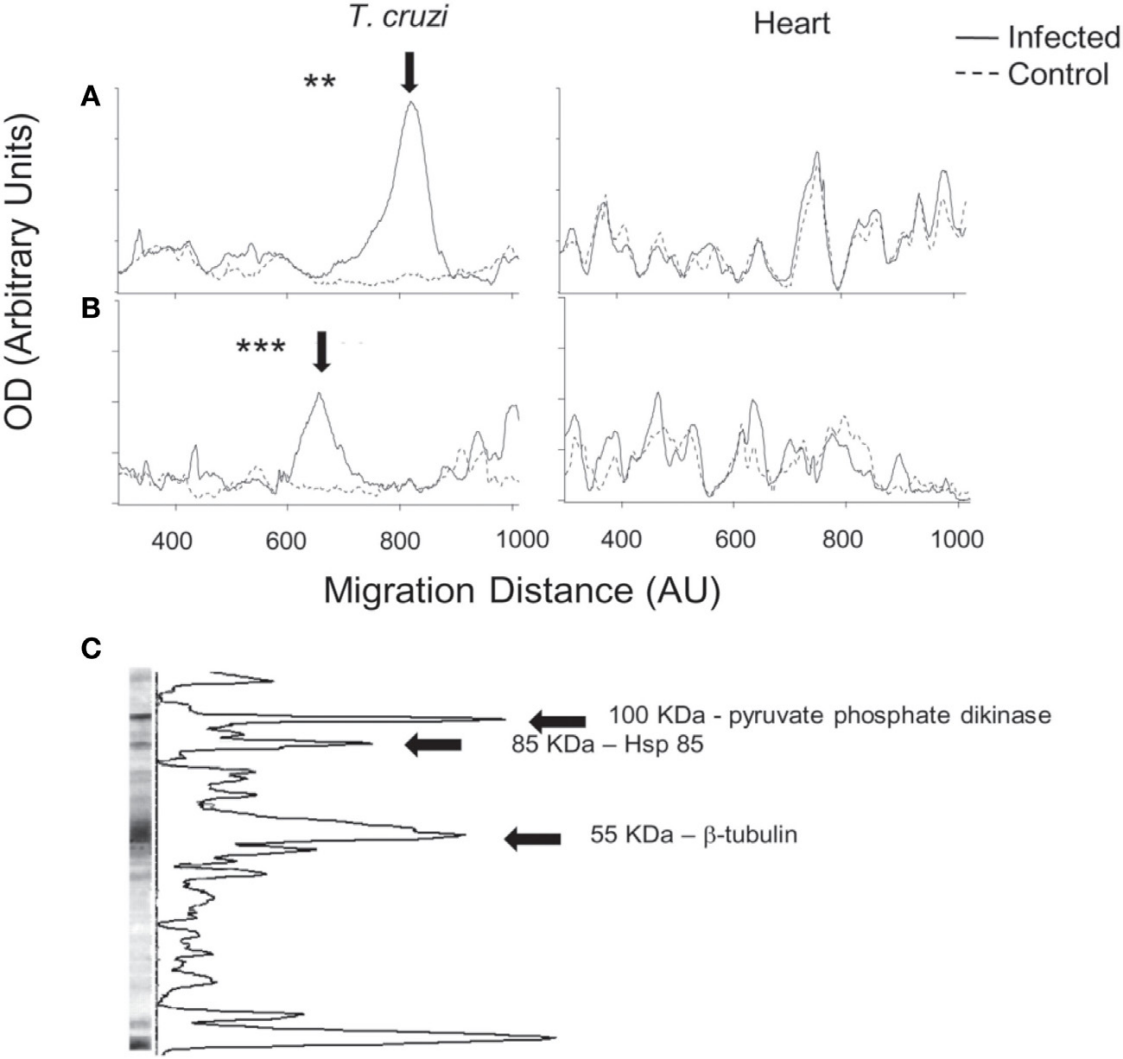
Global serum IgG reactivities against *Trypanosoma cruzi* and autologous heart polypeptides in infected BALB/c mice with *T. cruzi*. Mean densitometric profiles of IgG reactivities during acute **(A)** or chronic **(B)** infection on immunoblots against *T. cruzi* (left) and autologous heart polypeptides (right). Individual serum IgG concentrations were adjusted to 100 µg/mL. Arrow indicates the most prominent band of reactivity observed in the sera of acutely and chronically infected mice. Densitometric profiles represent the mean of 5–8 individual sera (see Materials and Methods) (**p < 0.01; ***p < 0.001). Immunoblots against heart polypeptides did not show significant differences. Data are representative of at least three independent experiments with five to eight mice per group. **(C)** A representative immunoblot of chronic serum against *T. cruzi* extract. Arrows indicate the three major *T. cruzi* protein bands, which were identified by mass spectrometry analysis as pyruvate phosphate dikinase (100 kDa), Hsp-85 (85 kDa), and β-tubulin (55 kDa). We could not identify the small-molecular weight band that also displayed a pronounced peak.

We analyzed global IgG antibody reactivities with a semiquantitative immunoblot technique against *T. cruzi* epimastigote extracts (13–16). Western blots of individual sera from chronic infection showed that IgG reactivities were directed to a restricted set of antigens (Figure 2A, right). The strongest reactivity was directed to a 50–55 kDa band, which was detected with sera from all infected mice (Figure 2A). This reactivity colocalized with the most abundant band present on Coomassie blue-stained gels of *T. cruzi* extracts (Figure 2B, left). We quantified the immunoblots from all animals at both acute and chronic infection and showed the average optical density profiles of the IgG reactivities against *T. cruzi* proteins (Figures 3A,B, left). Global IgG reactivity against *T. cruzi* was focused on a few bands of the parasite extract (Figures 3A,B, left). The most prominent antibody reactivity against *T. cruzi* was represented by a band located at positions 600 and 800 in the blots of acute and chronic stages, respectively, depending on conditions of the electrophoresis assay (arrows on Figures 3A,B, left). Reacting *T. cruzi* extract with a mixture of acute and chronic sera on the same membrane yielded a single band, suggesting that it was the same reactivity (not shown). Acutely infected *gld* mice gave the same prominent IgG reactivity (Figure S1 in Supplementary Material). In addition, experiments with trypomastigote enriched extracts indicated the presence of an intense 50–55 kDa IgG reactivity (data not shown).

Infection with *T. cruzi* results in myocarditis (18). We therefore analyzed IgG antibody reactivities against autologous heart polypeptides (13–16). Global IgG reactivity against cardiac polypeptides was not significantly increased following acute or chronic infection with *T. cruzi* (Figures 3A,B, right).

### Identification of *T. cruzi* β-Tubulin as a Major Antigenic Target for Antibodies in Infection

To identify the 50–55 kDa band reacting with sera from infection, the band was located in the gel with the help of the blot of a mirror lane, excised and subjected to mass spectrometry analysis. As shown in Table 1 and Figure 3C, the 50–55 kDa band was identified as *T. cruzi* β-tubulin (TcβTUB). We also identified two additional major *T. cruzi* antigens targeted by antibodies. The 100 kDa band was identified as pyruvate phosphate dikinase, and the 85 kDa band was identified as Hsp-85 (Figure 3C and data not shown).

**Table 1:** Mass spectrometry analysis of peptide fragments of the major 50–55 kDa band of *Trypanosoma cruzi* extract corresponding to *T. cruzi* β-tubulin.

Start^a^	End	Miss	Peptide sequence	Mr (expt)^b^	Mr (calc)	Delta	*m*/*z*^c^
310	318	0	R.YLTASALFR.G	1,040.5700	1,040.5655	0.0046	1,041.5773
253	262	0	K.LAVNLVPFPR.L	1,124.6709	1,124.6706	0.0003	1,125.6782
242	251	0	R.FPGQLNSDLR.K	1,145.5862	1,145.5829	0.0033	1,146.5935
381	390	0	R.VGEQFTAMFR.R	1,184.6026	1,184.5648	0.0378	1,185.6099
381	390	0	R.VGEQFTAMFR.R + Oxid. (M)	1,200.5578	1,200.5598	−0.0019	1,201.5651
242	252	1	R.FPGQLNSDLRK.L	1,273.6710	1,273.6779	−0.0068	1,274.6783
47	58	0	R.INVYFDEATGGR.Y	1,340.6339	1,340.6361	−0.0022	1,341.6412
381	391	1	R.VGEQFTAMFRR.K + Oxid. (M)	1,356.6472	1,356.6609	−0.0136	1,357.6545
63	77	0	R.AVLIDLEPGTMDSVR.A	1,614.8089	1,614.8287	−0.0198	1,615.8162
63	77	0	R.AVLIDLEPGTMDSVR.A + Oxid. (M)	1,630.7975	1,630.8236	−0.0261	1,631.8048
263	276	0	R.LHFFMMGFAPLTSR.G	1,653.7986	1,653.8160	−0.0174	1,654.8059
263	276	0	R.LHFFMMGFAPLTSR.G + Oxid. (M)	1,669.7885	1,669.8109	−0.0224	1,670.7958
263	276	0	R.LHFFMMGFAPLTSR.G + 2 Oxid. (M)	1,685.7819	1,685.8058	−0.0239	1,686.7892
263	276	0	R.LHFFMMGFAPLTSR.G + 2 Oxid. (M)	1,685.7819	1,685.8058	−0.0239	1,686.7892
337	350	0	K.NSSYFIEWIPNNIK.S	1,723.8311	1,723.8569	−0.0258	1,724.8384
47	62	1	R.INVYFDEATGGRYVPR.A	1,855.9092	1,855.9217	−0.0125	1,856.9165
363	379	0	K.MAVTFVGNNTCIQEMFR.R + Carbam. (C); Oxid. (M)	2,032.9008	2,032.9169	−0.0161	2,033.9081
104	122	1	K.GHYTEGAELIDSVLDVCRK.E + Carbam. (C)	2,161.0022	2,161.0474	−0.0451	2,162.0095
78	103	0	R.AGPYGQIFRPDNFIFGQSGAGNNWAK.G	2,811.2385	2,811.3517	−0.1132	2,812.2458
gi|18568139 beta-tubulin 1.9 (*T. cruzi*)							
Score: 105 sequence coverage: 36%^d^							

*^a^Peptide sequences and their corresponding “start” and “end” positions, as well as the number of missing cleavages are indicated*.

*^b^Expected and calculated molecular weight (in Da) for each peptide are shown; deltas are differences between expected and calculated values*.

*^c^Mass/charge ratio observed in MS spectrum (*m*/*z*)*.

*^d^The Mascot score for protein identification and the % of protein sequence coverage given by the identified peptides are also indicated*.

*Oxid. (M), methionine oxidation; Carbam. (C), *cys*-carbamidomethylation*.

**Figure 4:**
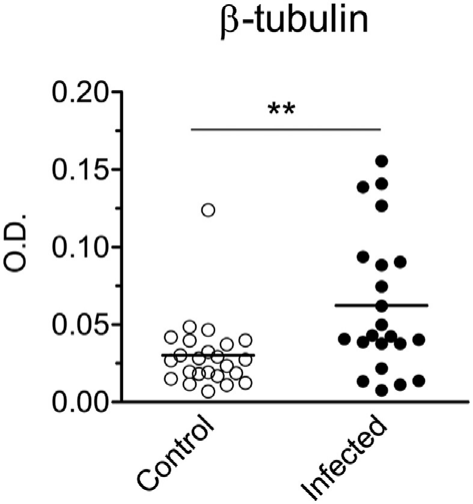
Infection with *Trypanosoma cruzi* increases IgG reactivity against *T. cruzi* β-tubulin. Serum IgG reactivities from control uninfected (open circles) or chronically infected (closed circles) BALB/c mice were assayed by ELISA on plates coated with recombinant TcβTUB. All IgG concentrations in sera were adjusted to 100 µg/mL. Each symbol corresponds to one individual mouse. Statistical analysis was performed by *t*-test from representative results of three similar experiments (**p < 0.01).

The *β-tubulin* coding region (19) was PCR amplified from *T. cruzi* Dm28c genomic DNA. The resulting fragment was cloned, sequenced and sub-cloned into pET-28a-TcβTUB expression plasmid. Recombinant TcβTUB was expressed and purified (Figure S2 in Supplementary Material). Following reaction with anti-β-tubulin monoclonal antibody, recombinant TcβTUB gave a single band compatible with the major band expressed in *T. cruzi* extracts (Figure 2B right). Analysis by ELISA confirmed that chronic infection with *T. cruzi* increased the production of IgG reactive with TcβTUB (Figure 4).

### Immunization With *T. cruzi* β-Tubulin Induced Protection Against Infection

**Figure 5:**
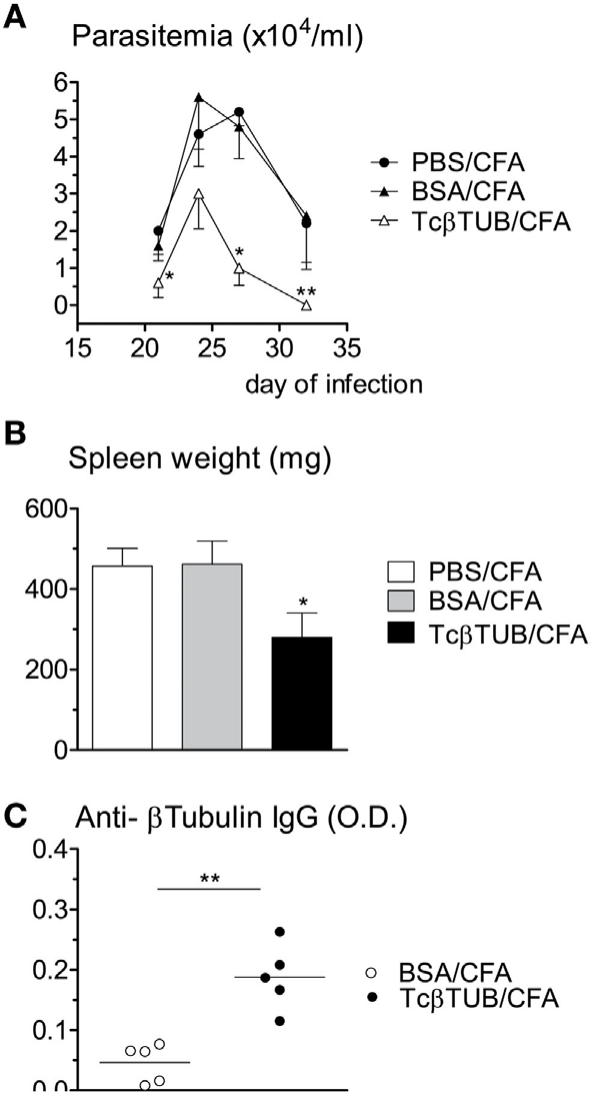
Immunization of naïve BALB/c mice with recombinant TcβTUB induces protection against *Trypanosoma cruzi* infection. **(A)** Kinetics of parasitemia. Groups of mice (*n* = 5 each) were immunized with PBS, BSA, or recombinant TcβTUB in CFA and were infected with *T. cruzi* Dm28c clone 14 days after immunization. Parasitemia was followed up to 32 days of infection. Data represent mean and SEM of groups. **(B)** Spleen weights of the same three groups after 32 days of infection. **(C)** After 32 days of infection, IgG levels against TcβTUB were determined for animals previously immunized with either BSA or TcβTUB in CFA. Statistical analysis was performed by two-way RM ANOVA test **(A)**, one-way ANOVA test **(B)**, and by *t*-test **(C)** from representative results of three similar experiments (*p < 0.05; **p < 0.01).

Naïve BALB/c mice were immunized with a single dose of recombinant TcβTUB in CFA and challenged with *T. cruzi* after 2 weeks. Control groups were immunized with PBS/CFA and BSA/CFA. All mice were sacrificed after 32 days of infection. Parasitemia was markedly reduced in mice immunized with TcβTUB/CFA, compared with control groups (Figure 5A). Spleen cellularity, which correlates with parasite burden in *T. cruzi* infection (32), was reduced in mice immunized with TcβTUB/CFA, compared with controls (Figure 5B). As expected, mice immunized with TcβTUB/CFA before infection produced higher amounts of anti-TcβTUB IgG, compared with controls (Figure 5C).

**Figure 6:**
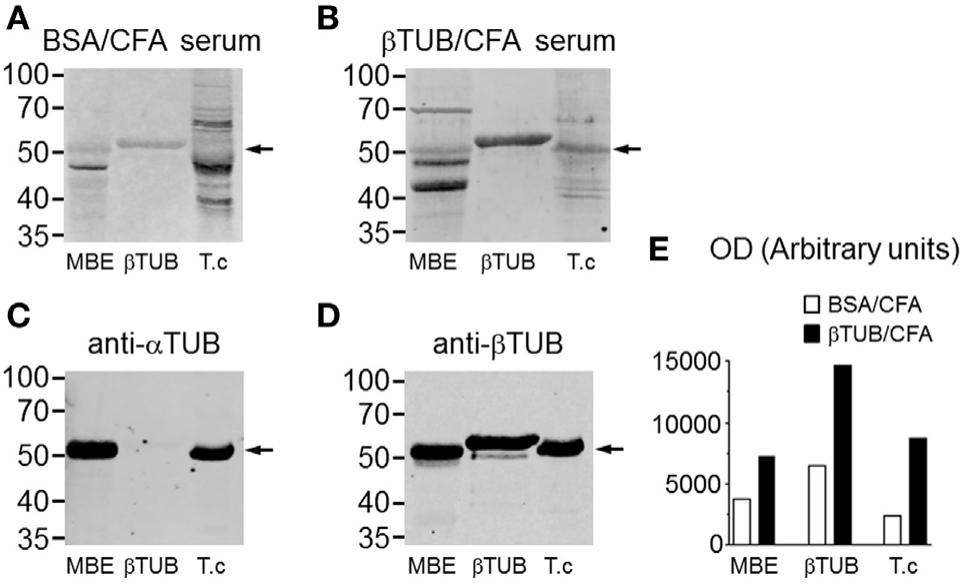
Increased IgG reactivity against β-tubulin in mice immunized with recombinant TcβTUB. **(A)** Immunoblots from mice immunized with BSA/CFA before infection and **(B)** mice immunized with TcβTUB/CFA before infection. Sera were blotted against mouse tubulin from mouse brain extract (MBE), recombinant TcβTUB and native tubulin from *Trypanosoma cruzi* extract. **(C)** Immunoblots of MBE, recombinant TcβTUB, and *T. cruzi* extract with monoclonal antibodies against α-tubulin; and **(D)** against β-tubulin. The shift in molecular weight of recombinant TcβTUB, compared with native mouse and *T. cruzi* β-tubulins, is due to an additional poly-histidine tail. **(E)** Densitometric profiles of the staining profiles presented in panels **(A,B)**.

IgG reactivities of control and immunized mice were compared by western blotting against mouse tubulin (MBE), recombinant TcβTUB, and native *T. cruzi* tubulin (Figure 6). IgG from mice immunized with BSA/CFA before infection reacted with mouse tubulin, TcβTUB and reacted weakly with native *T. cruzi* tubulin (Figure 6A). IgG from mice immunized with TcβTUB/CFA before infection reacted to TcβTUB and to native *T. cruzi* tubulin (Figure 6B). In addition, IgG from TcβTUB/CFA group showed increased reactivity to mouse tubulin (Figure 6B). These differences were confirmed by densitometric analysis and are shown in Figure 6E. Blots with monoclonal antibodies indicated the contents of α- and β-tubulin in mouse brain and *T. cruzi* extracts, confirmed that the recombinant antigen was β-tubulin, and identified the expected 2 kDa molecular weight shift due to an additional histidine tail in recombinant TcβTUB (Figures 6C,D). Taken together, our data on repertoire analysis indicated that TcβTUB is an immunodominant antigen in *T. cruzi* infection and that previous immunization with TcβTUB elicited a protective host immune response against infection.

## Discussion

Several proteins purified from *T. cruzi* induce protective immune responses. However, few studies identify the major protein antigens targeted by the antibody response of infected hosts. Here we investigated global changes in the antibody diversity of infected mice with *T. cruzi*. We employed a non-biased immunoblot technique against total parasite extracts. Under these selective conditions, the infection induced significant antibody reactivity against a range of *T. cruzi* antigens, which could be identified by proteomic analysis of the target proteins. We identified the major protein band recognized by IgG as TcβTUB. We cloned and expressed *T. cruzi* TcβTUB, and demonstrated that a single immunization with recombinant TcβTUB induced protection against *T. cruzi* infection. Therefore, our results indicated that repertoire analysis is a valid approach to identify new candidate antigens for a vaccine against Chagas disease.

Infection with *T. cruzi* can lead to polyclonal B lymphocyte activation, hypergammaglobulinemia, and production of autoantibodies (33, 34). We compared parasite-specific versus non-specific antibody reactivities elicited by infection. Since FasL-deficient mice undergo hypergammaglobulinemia, lymphoproliferation, and autoantibody production (35), we also compared antibody production by infected WT- and FasL-deficient *gld* mice. We used a limited set of exogenous and autologous antigens, and this approach did not reveal any evidence of autoreactivity or polyclonal lymphocyte activation. Our results might also reflect the use of Dm28c isolate, as parasite genetic diversity can influence the profile of immunoglobulins produced (36). On the other hand, age-matched FasL-deficient mice showed high levels of natural antibodies against *T. cruzi* and dsDNA, which did not increase following acute infection. Therefore, lymphocyte activation elicited by *T. cruzi* is weaker than that induced by the *gld* mutation. Chronic infection of *gld* mice increased the production of antibodies against *T. cruzi*, dsDNA and histone. The reason for increased responses to nuclear autoantigens is unknown but could be related to increased lymphoproliferation precipitated by *T. cruzi* infection. Production of antibodies against nuclear autoantigens correlates with increased apoptosis (37). Infection with *T. cruzi* exacerbates lymphocyte apoptosis (38, 39), which is partially dependent on the Fas/FasL death pathway (40–43), but apoptosis is also increased in *gld* mice (40).

Studies with purified *T. cruzi* proteins amplify the ability of antibodies to detect antigen by employing recombinant antigens or serum from immunized hosts (44). Instead, our approach employed immunoblots of whole *T. cruzi* extracts to detect antibody diversity of animals infected with *T. cruzi*. In this way, we identified by proteomic analysis the most immunogenic *T. cruzi* proteins serving as targets for the antibody response. A very limited number of protein bands reacted with the sera. The most prominent reactivity was a 50–55 kDa band which colocalized with the most abundant protein band of the *T. cruzi* extract. This band was excised, digested, analyzed by mass spectrometry, and identified as TcβTUB. All sera from infected mice reacted strongly with TcβTUB. In addition, in spite of already detectable immunological abnormalities, all sera from acutely infected *gld* mice reacted strongly with TcβTUB. These results suggest a robust response. Two additional bands recognized by host IgG antibodies were identified as *T. cruzi* pyruvate phosphate dikinase (100 kDa) and *T. cruzi* Hsp-85 (85 kDa).

We investigated IgG reactivity against autologous heart extracts. Global IgG antibody reactivity against heart polypeptides did not differ between control and infected sera. We detected increased or induced IgM reactivities against a set of cardiac polypeptides were following infection (data not shown). These cardiac polypeptides deserve further characterization.

To investigate the immunogenicity of TcβTUB, we cloned the *β-tubulin* gene from *T. cruzi* Dm28c genomic DNA and expressed recombinant TcβTUB in *E. coli*. Recombinant TcβTUB reacted with monoclonal antibodies against β-tubulin, but not α-tubulin. Chronic infection increased IgG reactivity to TcβTUB, compared with uninfected mice. Immunization of mice with a single dose of TcβTUB in CFA protected against *T. cruzi* infection, as seen by markedly reduced parasitemia, compared with animals immunized with PBS or BSA. Although our data show a wide range in the levels of anti-β tubulin antibody title in the immunized group, we observed a correlation of host protective response with higher IgG reactivity against recombinant TcβTUB. Mice immunized with TcβTUB before infection also gave increased reactivity against parasite and mouse tubulins.

Although *T. cruzi* tubulin reacts with antibodies during infection, to our knowledge it has not been tested previously as an immunogen. Interestingly, immunizations of mice with native *T. brucei* tubulin or recombinant *Trypanosoma evansi* beta-tubulin confer broad spectrum protection against infection by African trypanosomes (22, 23, 25). Humoral and cellular mechanisms leading to immunoprotection following immunization with TcβTUB are unknown. TcβTUB is expressed both intracellularly and at the surface of live parasites (45). The majority of monoclonal antibodies reacting against the membrane of live *T. cruzi* parasites recognize a 50/55 kDa antigen related to tubulin (46). In addition, antibodies reactive against parasite tubulin cross-react with host tubulin (47). Infection with *T. cruzi* increases the amount and the affinity of naturally occurring antibodies against autologous tubulin and induces novel specificities against tubulin fragments (48, 49). We found slightly increased reactivity against mouse tubulin in the serum of mice immunized with TcβTUB. Additional studies are required to determine whether increased humoral reactivity against autologous tubulin plays any deleterious effect in the host. Taken together, our results indicate that non-biased identification of immunodominant parasite antigens through analysis of antibody diversity of infected hosts is a valid approach to identify candidate antigens for vaccines against Chagas disease.

## Ethics Statement

This study was carried out in strict accordance with the recommendations in the Guide for the Care and Use of Laboratory Animals of the National Institutes of Health (USA). The protocol was approved by the Committee on the Ethics of Animal Experiments of the Health Science Center of the Federal University of Rio de Janeiro (CEUA-CCS, Permit Number: IBCCF 062/14), and all efforts were made to minimize suffering.

## Author Contributions

Conceived and designed the experiments: CGF-de-L, GADR, FM, and DON. Performed the experiments: FM, DON, CK, NH and MN. Analyzed the data: FM, DON, GADR, RV, CGF-de-L, and MN. Contributed reagents/materials/analysis tools: LML, PMB. RV, LF-de-L, AM, MFL, SMT. AM, NH, GD, and CGF-de-L. Wrote the manuscript: GD, MN, CGF-de-L, and FM.

## Conflict of Interest Statement

The authors declare that the research was conducted in the absence of any commercial or financial relationships that could be construed as a potential conflict of interest.
