# Supplementary figures and images for: Antibody Repertoires Identify β-Tubulin as a Host Protective Parasite Antigen in Mice Infected With Trypanosoma cruzi
Source: Front Immunol. 2018 Apr 13;9:671. doi: 10.3389/fimmu.2018.00671 (PMC5909033; doi:10.3389/fimmu.2018.00671)

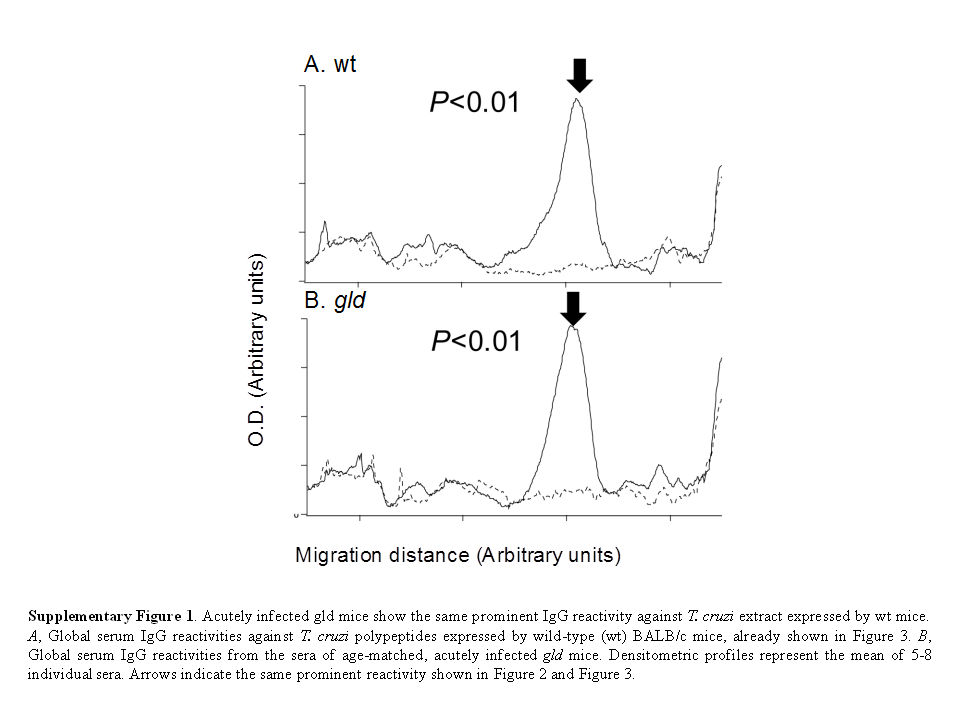

Supplement: Supplementary file 1 [file Image_1.tif]

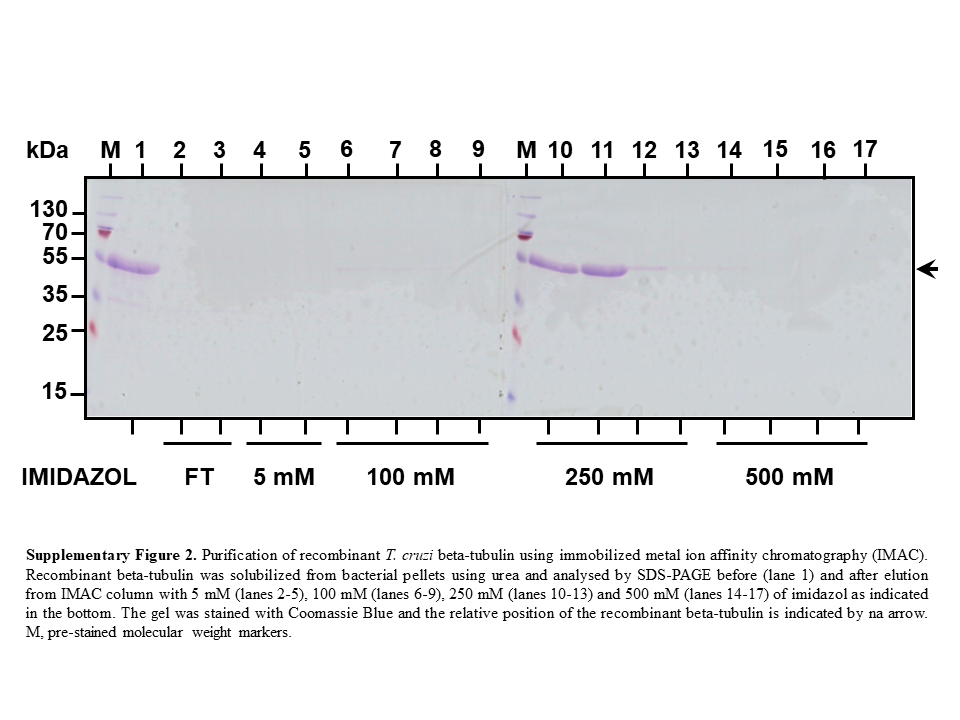

Supplement: Supplementary file 2 [file Image_2.tif]
